# Human platelet lysate as a substitute for serum in natural killer cell generation and expansion

**DOI:** 10.1093/lifemedi/lnad011

**Published:** 2023-03-13

**Authors:** Chao Li, Hao Zhu, Lei Zhang, Xianwei Liu, Yibing Ji, Haihong Zhang, Zhongpeng Li, Chen Wu, Fangfang Zhu

**Affiliations:** HemaCell Biotechnology Inc., Suzhou 215123, China; HemaCell Biotechnology Inc., Suzhou 215123, China; HemaCell Biotechnology Inc., Suzhou 215123, China; HemaCell Biotechnology Inc., Suzhou 215123, China; HemaCell Biotechnology Inc., Suzhou 215123, China; HemaCell Biotechnology Inc., Suzhou 215123, China; HemaCell Biotechnology Inc., Suzhou 215123, China; HemaCell Biotechnology Inc., Suzhou 215123, China; HemaCell Biotechnology Inc., Suzhou 215123, China; School of Biomedical Engineering, Shanghai Jiao Tong University, Shanghai 200030, China

**Dear Editors,**

Natural killer (NK) cells are increasingly being recognized as a promising cellular tool for cancer immunotherapy [1, 2]. Producing clinical-grade cells is therefore essential for NK cell-based immunotherapies. Currently, several differentiation and expansion protocols for NK cells derived from induced pluripotent stem cells (iPSCs) or hematopoietic stem cells (HSCs) have been developed, and the efficacy of these NK cells have been demonstrated in clinical trials [3–5]. However, these differentiation/expansion protocols commonly employ human AB serum (ABS) as a medium supplement, which has unknown and variable components [6]. This undefined composition poses significant issues related to cellular heterogeneity and may not be sufficient to meet the expected purity for clinical-grade cell therapies. Therefore, it is critical to identify a substitute for ABS in NK generation and expansion. In this study, we investigate whether human plasma lysates (hPL) can be used as a substitute for ABS in producing NK cells from human CD34^+^ HSCs using a two-stage differentiation protocol (Fig. 1A). In the first stage, CD34^+^ cells were cultured in NK differentiation medium containing cytokines (IL-3, IL-7, IL-15, SCF, FLT3L) for 8 days; in the second stage, IL-3 was removed from the medium, and cells were cultured for another 22 days until a near pure NK population was generated. NK cells could be further expanded after Stage 2 for 2–3 weeks. hPL or ABS was added to the culture in the differentiation and expansion stages. We found that both hPL and ABS conditions were able to produce phenotypically mature NK cells (Fig. 1B and 1C). To evaluate any differences between hPL and ABS in NK derivation, we measured cell proliferation and viability during differentiation. Our results showed no significant difference of cell viability was observed between hPL-induced and ABS-induced conditions (Fig. 1D). Instead, we found that hPL actually significantly promoted cell proliferation compared to ABS, starting from day 17 of differentiation (Fig. 1E). Interestingly, we observed that a large population of NK cells was generated on days 14–17 during differentiation. This indicated that hPL facilitated NK cell proliferation during differentiation, to a greater extent than ABS, while maintaining comparable cell viability. Meanwhile, we also monitored the differentiation process with flow cytometry for typical NK cell markers. We began to observe CD56 expression from day 10 of differentiation in both conditions, and the CD3^−^CD56^+^ population increased throughout the differentiation (Fig. 1F). At day 30, almost all HSC-derived cells expressed CD56, while some cells also expressed natural cytotoxicity receptors CD335 and CD336, in both hPL and ABS conditions (Fig. 1G and 1H). No T-cell marker CD3 was detected. Interestingly, we observed a higher population of CD56^+^CD16^+^ in the hPL group (21.9%) than in the ABS group (3.42%) as well as an increased expression of CD335 in the hPL group (57%) than in the ABS group (17.1%), indicating that hPL might benefit the generation of mature and cytotoxic NK cells (Fig. 1G and 1H). These results suggested that hPL is a suitable or even better medium supplement than ABS in NK derivation.

**Figure 1.**
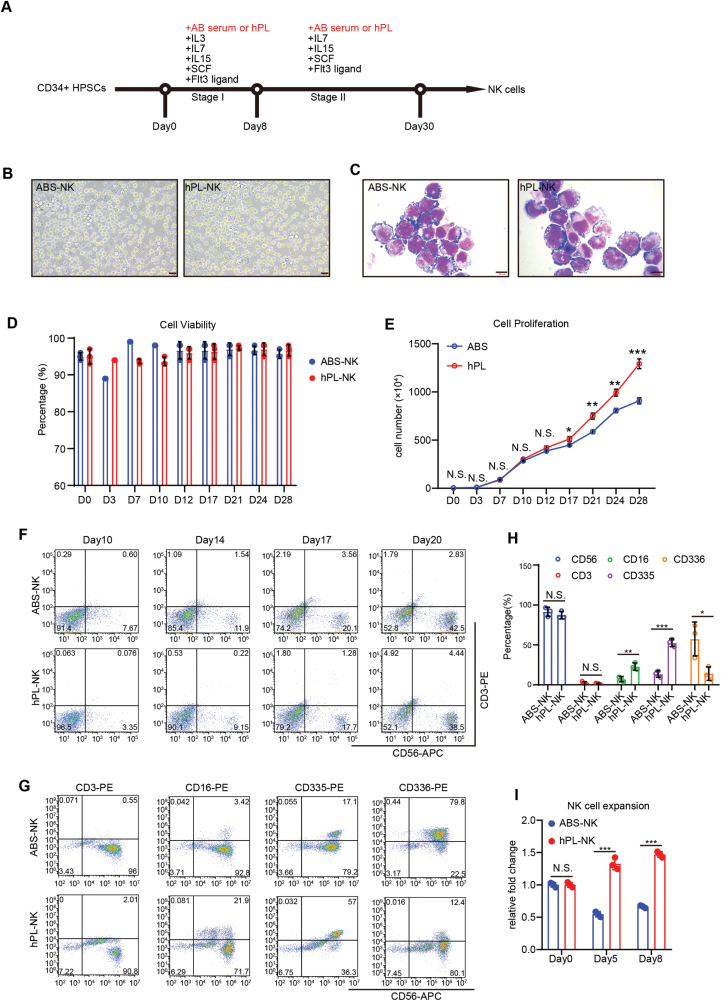
hPL can effectively induce the differentiation of CD34^+^ HSCs into NK cells. (A) Schematic diagram of *in vitro* NK cell differentiation from CD34^+^ HSCs. (B) Phase images of differentiated NK cells on day 12 in the present of ABS (ABS-NK) and hPL (hPL-NK). Scale bar = 20 μm. (C) Giemsa staining of ABS-NK and hPL-NK on day 10. Scale bar = 10 μm. (D) Cell viability of NK cells during the 28 days of differentiation in ABS and hPL groups. The mean values ± SD are shown. *n* ≥ 3. (E) Growth curve of NK cells in 28 days of differentiation in ABS and hPL groups. The mean values ± SD are shown. *n* ≥ 3. N.S., no significant difference, *P < 0.05, **P < 0.01, ***P < 0.001. (F) The generation of NK cells (CD3^−^CD56^+^) during differentiation in both ABS and hPL groups. (G) ABS-NK cells and hPL-NK cells were stained for a panel of NK cell receptors on day 30. Expression of each marker is shown by representative flow cytometry plots. (H) Statistics of expression level of each marker in Fig. 1G. The mean values ± SD are shown. *n* ≥ 3. *P < 0.05, **P < 0.01, ***P < 0.001, N.S., no significant difference. (I) Expansion of ABS-NK and hPL-NK after differentiation. Relative fold change means log2 (fold change) relative to day 0. The mean values ± SD are shown. Significance is calculated by Student’s *t* test. ***P < 0.001, N.S., no significant difference.

Previous studies have indicated that hPL promotes the proliferation of various cell types, such as mesenchymal stem cells [7]. In this study, we wanted to investigate the effect of hPL on the expansion of HSC-derived NK cells. To do this, we first differentiated CD34^+^ cells for 28 days until a pure CD56^+^ NK cell population was produced. We then cultured these HSC-derived NK cells in hPL or ABS conditions and expanded them in the absence of feeder cells. Cell number was recorded at different time points. As shown in Fig. 1I, NK cells could be expanded in both conditions, which is consistent with previous studies with primary NK cells [8, 9]. Interestingly, a more significant cell proliferation rate was observed in hPL group compared to the ABS group. These results suggested that hPL might be a better supplement for NK cell expansion and therefore could be used to facilitate clinical-scale NK production.

To further characterize the effects of hPL, we performed killing assays with the HSC-derived NK cells, since cytotoxicity is the most important function for NK cells (Fig. 2A). The cytotoxic activity of both hPL-induced and ABS-induced NK cells (hPL-NK and ABS-NK) was assessed in killing assays using three tumor cell lines K562, HepG2 or A549 as targets. K562 cells were first co-incubated with NK cells at different effector-to-target ratios (5:1 and 10:1) for 3.5 h and then measured by flow cytometry. The results showed no significant differences between hPL-NK and ABS-NK (Fig. 2B) using this tumor cell line. The IncuCyte real-time killing assay against HepG2 also exhibited comparable results (Fig. 2C). Interestingly, in the A549 tumor cell line, hPL-NK showed comparable killing to ABS-NK at the low E/T ratio (2:1), while better killing activity by hPL-NK was observed at the higher E/T ratio (4:1) (Fig. 2D). Taken together, the hPL-NK showed a comparable or better cytotoxicity activity than ABS-NK across the different tumor cell lines.

**Figure 2.**
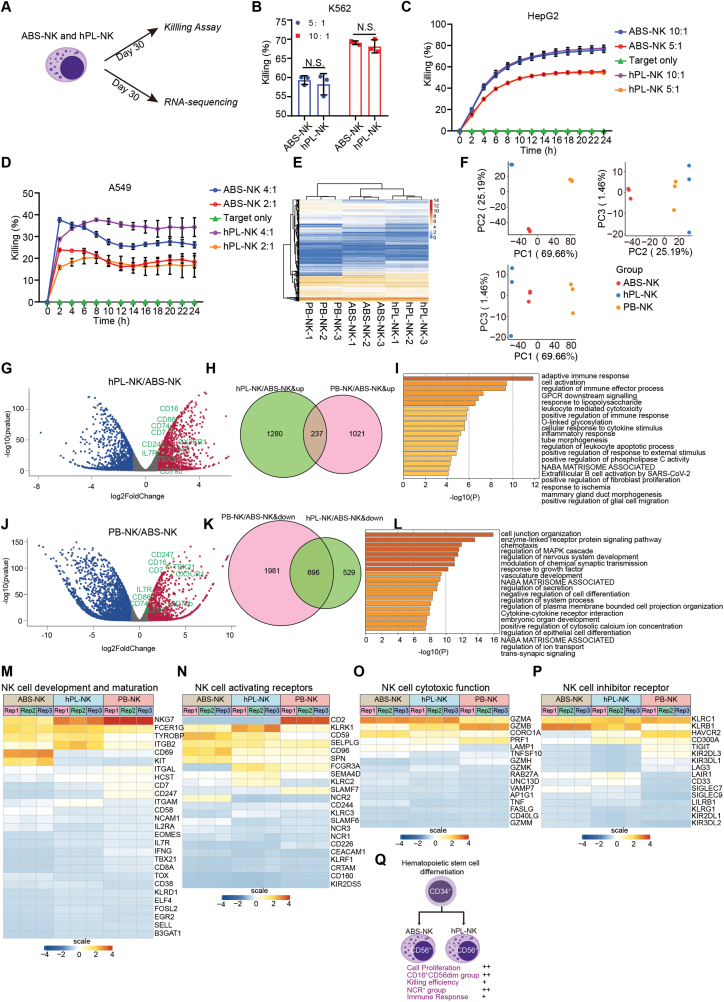
Human platelet lysates benefit the maturation, development, and cytotoxicity of NK cells. (A) The schedule of NK cell functional assay and mechanism study. (B) *In vitro* killing against K562 cells (effector: target ratio from 5:1 to 10:1) for 3.5 h. The mean values ± SD are shown, *n* ≥ 3. N.S., no significant difference. (C) *In vitro* killing against HepG2 cells (effector: target ratio from 5:1 to 10:1) for 24 h. The mean values ± SD are shown. *n* ≥ 3. (D) *In vitro* killing against A549 cells (effector: target ratio from 2:1 to 4:1) for 24 h. The mean values ± SD are shown. *n* ≥ 3. (E) Unsupervised hierarchical clustering of ABS-NK, hPL-NK, and PB-NK. Three replicates were analyzed for each condition. (F) Principal component analysis (PCA) of ABS-NK, hPL-NK, and PB-NK. (G) Volcano plot representing differentially expressed genes in hPL-NK/ABS-NK. Red dots represent up-regulated genes and blue dots represent down-regulated genes. (H) Venn diagram representing the up-regulated genes in groups of hPL-NK/AB-NK (Green) and PB-NK/ABS-NK (Pink). (I) Gene ontology functional enrichment (GO) of the overlap genes in Fig. 2H. (J) Volcano plot representing differentially expressed genes in PB-NK/ABS-NK. Red dots represent up-regulated genes and blue dots represent down-regulated genes. (K) Venn diagram representing the down-regulated genes in groups of hPL-NK/ABS-NK (Green) and PB-NK/ABS-NK (Pink). (L) Gene ontology functional enrichment (GO) of the overlap genes in Fig. 2K. (M) Heatmap of genes associated with development and maturation of NK cells. Rep1, Rep2, Rep3 represents three biological replicates. (N) Heatmap of activating receptor of NK cells. (O) Heatmap of genes associated with cytotoxic function of NK cells. (P) Heatmap of inhibitory receptor of NK cells. Heatmaps were generated using Z scores derived from transformed RNA-seq counts using regularized-logarithm transformation (rlog). Each column represents a biological replicate. (Q) Schematic summary of the comparison between hPL and ABS in NK cell phenotyping, function, and gene expression profile.

To investigate the potential mechanism of hPL in differentiation and expansion of NK cells, we employed RNA-sequencing analyses to explore the transcriptomes of hPL-NK, ABS-NK, and peripheral blood-isolated primary NK cells (PB-NK). Cluster analysis of whole transcriptome levels showed that all three groups clustered closely in gene expression patterns (Fig. 2E). Interestingly, principal components analysis (PCA) showed that hPL-NK and ABS-NK are similar on PC1/PC2 and PC1/PC3 which is consistent with gene expression patterns that hPL-NK and ABS-NK were relatively closer on global transcriptome (Fig. 2F). However, we found that PB-NK were much more similar to hPL-NK in PC2/PC3 compared to ABS-NK which indicated that hPL-NK shared some patterns with PB-NK (Fig. 2F). In addition, a total of 1517 up-regulated genes were discovered in hPL-NK compared to ABS-NK, while 1258 up-regulated genes were found in PB-NK compared to ABS-NK, among which 237 genes overlapped (Fig. 2G–J). These 237 up-regulated genes represent the shared targets that were activated in both hPL-NK and PB-NK comparing to ABS-NK. Gene ontology (GO) analysis of these 237 overlapping genes illustrated that immune related pathways were activated in hPL-NK and PB-NK rather than ABS-NK (Fig. 2I) which indicated that hPL-NK and PB-NK shared activated immune pathways that might affect NK function. Similarly, GO analysis of 696 down-regulated genes overlapping in hPL-NK vs ABS-NK and PB-NK vs ABS-NK showed that non-related pathways and negative regulation of immune pathways were inhibited in hPL-NK and PB-NK (Fig. 2G and 2J–L) which suggested that the down-regulated genes are not related to NK function. To further investigate the effect of hPL, we analyzed differentially expressed genes related to NK activating receptors, inhibitory receptors, cell development and maturation, and cytotoxic function. The heatmap showed that the expression of these genes in hPL-NK were closer to that in PB-NK, particularly in cell development and maturation, and cytotoxic function groups (Fig. 2M–P). These results indicated that hPL might have a better effect in promoting NK cell maturation and improving NK cytotoxicity than ABS.

In summary, our work evaluated the feasibility of using hPL as a serum replacement in the production of NK cells. We demonstrated that hPL is suitable as a medium supplement for NK cell generation, maturation, and proliferation, comparable to or better than human ABS. The phenotype of induced NK were conserved in conditions with hPL and ABS. Moreover, hPL showed a comparable or even better effect on cell proliferation and viability than ABS during differentiation. Interestingly, hPL increased the CD56^+^CD16^+^ NK population, indicating that hPL benefits NK maturation and might have a positive effect on NK cell cytotoxicity. More evidence was found in differential gene expression analysis to support this notion. hPL-NK showed a closer gene expression pattern of cell development and maturation to PB-NK than ABS-NK. Further cytotoxicity assessments showed that hPL-NK and ABS-NK exhibited similar cytotoxicity profiles against K562 and HepG2 cell lines, which is consistent with the RNA-sequencing results showing that the transcriptional level of inhibitory receptors and activating receptors were similar in hPL-NK and ABS-NK. Additionally, we found that hPL-NK showed an increased killing activity against A549 compared to ABS-NK, which also suggested that hPL has a positive effect on NK cytotoxicity against certain tumor types.

## Research limitations

This study has two research limitations that should be addressed in future studies. While hPL showed positive effects on NK generation and short-term expansion, the effect of hPL on long-term expansion has not been evaluated. It has been reported that higher expansion rate and better viability was observed in NK cell culture with hPL [10]. This might help explain the use of hPL on NK expansion. Moreover, we found that hPL-NK exhibited increased expression of maturation and cytotoxic-related genes. However, the killing assay results only showed a difference in the A549 tumor cell line, while there was no significant cytotoxicity difference in K562 or HepG2 cells. This differential effect of cytotoxicity on tumor cell lines also needs to be further investigated.

## Supplementary Material

lnad011_suppl_Supplementary_Material
