# Supplementary material for: Human platelet lysate as a substitute for serum in natural killer cell generation and expansion
Source: Life Med. 2023 Mar 13;2(2):lnad011. doi: 10.1093/lifemedi/lnad011 (PMC11749240; doi:10.1093/lifemedi/lnad011)
Supplement: lnad011_suppl_Supplementary_Material [file lnad011_suppl_Supplementary_Material.docx]

**Supplementary Materials**

Human platelet lysate as a substitute for serum in natural killer cell generation and expansion

**Material and Method**

**NK cell derivation from CD34^+^ HSCs**

CD34^+^ HSCs (Miaoshun Biotechnology Co., Ltd) were seeded in plates with NK differentiation basic medium (56.6% DMEM medium with GlutaMAX™, 28.3% F12 medium with GlutaMAX™, 10% hPL(Helios) or 15% human AB serum (Sigma), 1% P/S, 2 mM L-glutamine, 25 uM β-mercaptoethanol, 50 uM ethanolamine) with the cytokine cocktail including IL-3 (5 ng/mL), IL-15 (10 ng/mL), IL-7 (20 ng/mL), SCF (20 ng/mL) and FLT3 ligand (10 ng/mL). After 8 days of differentiation, cells were transferred into NK differentiation basic medium with another cytokine cocktail including IL-7, IL-15, SCF and FLT3 ligand. Half-medium changes were performed every 2–3 days. NK cells were harvested at indicated time points for analysis.

**Flow Cytometry**

Flow cytometry was done on a Beckman CytoFLEX, and data were analyzed using FlowJo. The following antibodies were used: CD56-APC (1:50), CD3-PE (1:50), CD16-PE (1:50), CD314-PE (1:50), CD335-PE (1:50) and CD336-PE (1:50). All antibodies were purchased from BioLegend.

***In vitro* cytotoxicity assays**

K562 were incubated with CFSE for 15 min at 4℃, washed 3 times, and then cocultured with NK cells at the indicated effector to target ratios. After 3.5 hours of incubation, cells were harvested and stained with PI for 5min, and then analyzed by flow cytometry. HEPG2 or A549 cells were cocultured with NK cells at the indicated effector to target ratios in E-plate for 50 hours and then cell index was quantified using xCElligence RTCA SP system.

**RNA-sequencing**

After 30 days of differentiation with hPL or ABS, NK cells were harvested. Along with peripheral blood-isolated NK cells, total RNA of three groups was isolated and purified using TRIzol (Invitrogen).Total RNA was then sent to Geekgene Technology for RNA-sequencing. RNA-sequencing libraries were constructed using KAPA Hyper Prep kits (KK8504). The libraries were sequenced with Illumina Novaseq 6000. Sequence reads were trimmed for adaptor sequence/low-quality using Trimmomatic (parameter - Quality limit : LEADING:3 TRAILING:3 SLIDINGWINDOW:4:15 MINLEN:36). Trimmed sequence reads were mapped to hg19 using STAR (parameters-mismath cost:--alignIntronMax 100000 --chimSegmentReadGapMax parameter 3 --alignSJstitchMismatchNmax 5 -1 5 5). Read count extraction and normalization were performed using FeatureCounts and Stringtie. Tab-delimited text files include FPKM value for each Sample. KEGG functional analysis was performed using the R package clusterProfiler. GO functional analysis was performed using the R package topGO. The R package DESeq2 was used for differential expression analysis.

**Statistical analysis**

Data are presented as the mean plus or minus standard deviation. The significance of differences between groups was determined by two tailed unpaired *t*-test in GraphPad Prism 8. *P*-values < 0.05 were considered statistically significant.

**Data availability**

The data supporting the findings of this study are available within the article or its supplementary materials.

**Research ethics**

This research was not involved human subjects and animal experiment.
